# Optimization of Ultrasound-Assisted Extraction of Polyphenol Content from *Zea mays* Hairs (Waste)

**DOI:** 10.1155/2020/5072938

**Published:** 2020-12-17

**Authors:** Sarra Aourabi, Mouhcine Sfaira, Fatima Mahjoubi

**Affiliations:** Laboratory of Engineering, Modeling and Systems Analysis (LIMAS), University of Sidi Mohamed Ben Abdellah (USMBA), Faculty of Sciences, PO Box 1796-30000, Fez-Atlas, Morocco

## Abstract

The aim of this study was to achieve the best extraction efficiency of the hydroethanolic extract of *Zea mays* hairs. The impacts of ethanol concentration, extraction time, and solvent /material ratio were studied in relation to the performance of *Zea mays* extracts by ultrasonic extraction at 50 kHz and room temperature. All extracts were quantitatively characterized in terms of polyphenol content. Response surface methodology (RSM) was carried out to optimize the extraction process and increase extraction efficiency. In the experiments, different concentrations of ethanol:water were used. The efficiency of the extraction process was determined from an analysis of variance (ANOVA). The maximum extraction efficiency of the hydroethanolic extraction (31.37%) and the quantitative value of the polyphenol content (257.87 mg EAG/g extract) were obtained using a treatment time of 40 min, an ethanol:water (70 : 30), and a solvent/material ratio (11 mL/g). The results obtained indicate that ultrasonic-assisted extraction is an effective method for extracting natural compounds from *Zea mays*, thus allowing the full use of this abundant and inexpensive industrial waste.

## 1. Introduction

*Zea mays* hairs (*Z. mays*, corn silk) are a collection of the stigmas (fine, soft, yellowish threads) from the female flowers of the maize plant contains proteins, vitamins, carbohydrates, Ca^2+^, K^+^, Mg^2+^ and Na^+^ salts, volatile oils, and steroids, alkaloids, saponins, tannins, flavonoids, flavones, and flavone glycosides [1, 2]. Also, *Z.mays* hairs, as an essential part of maize, have high pharmaceutical values, for example, it is a urinary emulsifier, which passes stones and gravel from the kidneys and bladder; these hairs work against benign prostatic hyperplasia, cystitis, gout, chronic nephritis, and other similar diseases [3]. In previous studies, a series of phenolic compounds such as rutin, quercetin, epicatechin, vanillic acid, gallic acid, and flavone have been identified and isolated from *Z. mays* hairs [4–6]. Polyphenols (containing at least two phenolic groups (hydroxybenzenes)) are present in most plant foods and have been considered as “antinutrients” [7, 8]. Pharmacological studies have shown that phenolic compounds have an important function in human health [9], including anticancer actions [3, 10] and antioxidant activity [11]. Conventional extraction methods such as maceration, decoction, infusion, reflux, heating, and Soxhlet extraction are used to extract biologically active polyphenols from various plants. However, these conventional extraction methods have generally many disadvantages such as large amount of solvent utilization, long extraction time, and lower extraction yield [12, 13]. In recent years, new extraction techniques such as ultrasonic assisted extraction (UAE) [14], microwave assisted extraction (MAE) [15], and accelerated solvent extraction (ASE) [16] have been developed to increase the efficiency of the extraction process in terms of time, solvent consumption, and energy consumption [17–20]. Among these methods, the UAE method is advantageous for polyphenol extraction because of the simplicity of the process, low working temperatures (35°C to 45°C), low solvent consumption rate, high recovery of polyphenolic compounds, and low energy loss [21]. Polyphenols are highly soluble in various solvents. Methanol and acetone have toxic effects on the human body; in addition, the cost of production is high, so ethanol is still used as an extraction solvent because of its nontoxicity and low-cost availability. The response surface methodology (RSM), especially the Box–Behnken design method, is a modeling tool used to optimize extraction conditions by evaluating multiple parameters and their interaction effects from quantitative data. As a result, RSM can not only statistically optimize complex extraction procedures but also reduce the number of experimental trials [22]. RSM has often been used to optimize the extraction of phenolic compounds from different plant sources [23]. In this work, three factors were used to study the effects of certain parameters on extraction efficiency and the level of polyphenols contained in *Z. mays* hairs (waste), namely, ethanol concentration, extraction time, and solid/liquid ratio.

## 2. Experimental

### 2.1. Vegetal Material

Female *Z. mays* hairs, Poaceae, used for this investigation were collected in September 2018 at the region of Taounate. The botanical identification of the species was carried out in the Laboratory of Biotechnology and Preservation of Natural Resources (BPNR), University Sidi Mohamed Ben Abdellah, Fez, Morocco. The *Z. mays* hairs were dried at room temperature.

### 2.2. Method of Extraction

The extracts of the *Z. mays* hairs were obtained by ultrasounds (45 Hz, 50 W, 308 K). The extraction was performed at different ethanol/water percentages, for the amount of material, and for different periods. The extracts were filtered, concentrated under reduced pressure.

### 2.3. Effect of Operating Parameters on Extraction Yield

The fixed extraction conditions are as follows: ultrasonic time 45 min, solvent-to-material ratio 10 : 1, ethanol concentration 70%, ultrasonic temperature 35°C, and ultrasonic power 50 W. After that, we keep one variable fixed and change the others. The range of variance for each variable is (35 min, 40 min, 45 min,50 min, and 55 min) for the extraction time, (5 : 1, 10 : 1, 15 : 1, 20 : 1, and 25 : 1) for the liquid material ratio, and (40, 50, 60, 70, and 80%) for the ethanol concentration. Their impact on the extraction efficiency of the materials was tested separately.

### 2.4. Experiment Design for Optimization of Extraction Method

The RSM is a statistical method that uses quantitative data from an appropriate experimental design to determine the optimal conditions for extraction; first of all, the factors that influence extraction yields and total phenol content were determined and variance ranges of each independent variable were obtained. The results showed that three main factors influence the extraction efficiency, including ethanol concentration (%, *X*1), solvent/material ratio (mL/g, *X*2), and extraction time (W, *X*3), and these factors were used as independent variables to optimize extraction conditions. We determined the factors and their ranges of variables based on the experiments on the single factors and found that the range of variance for each factor was as follows: ethanol concentration (50% to 70%) and solvent/material ratio (10 mL/g to 20 mL/g). Temperature was not taken into account in this study since the sample was treated at 35°C to avoid degradation of heat-sensitive polyphenolic compounds. In this study, the experiments were carried out on the basis of a Box–Behnken design (BBD) plan. The coded values of the experimental factors are indicated in Table 1. The complete design included 26 combinations, with two repetitions at the center point (Table 2). The data from BBD were analyzed by multiple regressions to fit the following quadratic polynomial model equation:

(1) $$\begin{array}{c} Y=\beta_{0}+\sum b_{i}X_{i}+\sum b_{ii}X_{i}^{2}+\sum b_{ij}X_{i}X_{j}, \end{array}$$

where ***Y*** is the predicted response, *ß*_0_ is a constant, and *b*_*i*_, *b*_*ii*_, and *b_ij_* are the linear, quadratic, and interactive coefficients of the model, respectively. Accordingly, *X*_*i*_ and *X*_*j*_ represent the levels of the independent variables, respectively. The quality of the fitted model was expressed with the coefficient of determination (*R*^2^).

### 2.5. Determination of the Total Phenolic Content

The total phenols of the *Z. mays* hair extract were estimated using the Folin–Ciocalteu method [24].

### 2.6. Statistical Analysis

The experimental results of the response surface design were analyzed by Nemrodw. The modeling was started with a quadratic model, including linear, squared, and interaction terms. Significant terms in the model for each response were found by analysis of variance (ANOVA). The experimental data were evaluated with descriptive statistical analyses such as *p* value. The *p* values <0.05 were considered to be statistically significant. All experiments were conducted in triplicate unless otherwise noted in the text. The purpose of statistical analysis is to determine the different parameters (determination coefficient *R*^2^ and the effects of the factors) in order to determine the statistical significance of the factors studied.

## 3. Results and Discussion

### 3.1. Effect of Single Factor on the Extraction Yield of *Z. mays* Hairs

#### 3.1.1. Effect of Extraction Time

**Figure 1:**
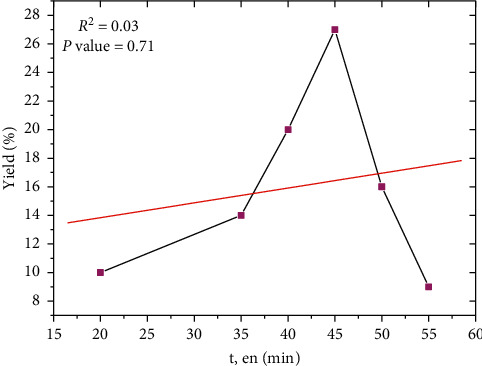
Effects of extraction time on yield of extraction.

The results in Figure 1 indicate that the highest extraction efficiency increases with time and reaches its maximum value at 45 min, but after 45 min, the efficiency decreases. However, when the extraction time is extended, all the cells of the plant will be completely cracked due to cavitation effects, and therefore, the extraction yield increases. Therefore, when plant cells decompose, various compounds such as insoluble substances are suspended in the extraction liquid, which reduces the permeability of the solvent [25]. Therefore, polyphenol yield is reduced due to the reabsorption of target components on ruptured tissue particles due to their relatively large specific surface area [26].

#### 3.1.2. Effect of Ethanol Concentration

**Figure 2:**
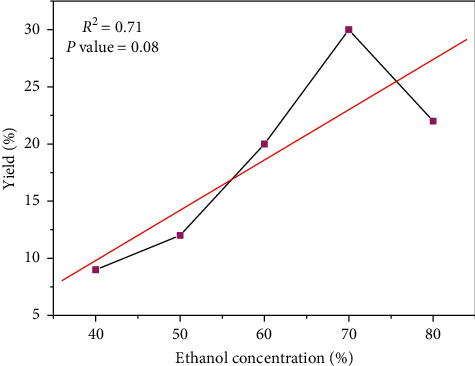
Effects of ethanol concentration on yield of extraction.

Water as a nontoxic and inexpensive solvent has widely been used for extraction of active components. It has been observed that sometimes the extraction yield could be improved by adding small percentages of water to the extraction solvent [27]. According to Figure 2, the extraction efficiency of polyphenols has gradually increased to a maximum of 70%. Then, the yield dropped sharply, due to increased volatilization with a high concentration of ethanol.

#### 3.1.3. Effect of Solvent-to-Material Ratio

**Figure 3:**
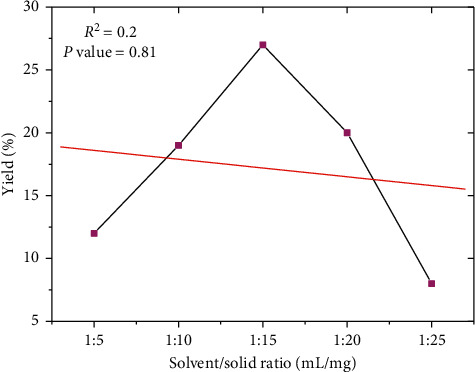
Effect of solvent-to-material ratio on yield extraction of extraction.

The maximum polyphenol yield was obtained at a ratio of 15 : 1 between liquid and material (Figure 3). This was consistent with the principle of mass transfer, according to which the driving force during mass transfer was the concentration gradient between solid and liquid, which increases when a higher solvent-to-sample ratio is used [28, 29]. However, an excessive increase in the ratio has also resulted in a decrease in extraction efficiency [30].

### 3.2. Statistical Analysis and Model Fitting of Yield Extraction

The yield of extraction of the experimental design is presented in Table 2. The decoded values of independent variables in the experiment were also presented. Yield extraction of hydroethanolic extract varied from 9.31 to 27.34% based on different extraction conditions. Multiple regression analysis of variance (ANOVA) was used to check the adequacy and fitness of the developed models. The results of ANOVA are given in Table 3. The results showed that the response of the worth rate (*Y*) model was highly significant. An item ethanol concentration (*X*1), solvent-to-material ratio (*X*2), and time of extraction (*X*3) were significant. The second-order items reached extremely significant level (Table 4). The *R*^2^ and *R*^2^_Adj_ values were 0.977 and 0.964, respectively. The values are close to 1 which means that the module is validated. The *R*^2^_pred_, which is a measure of how a particular model fits each point in the design, was 0.941, shown strong correlation with actual experimental values.

To calculate the yield of extraction (*Y*1) encoded by independent variable ethanol concentration (*X*1), solvent-to-material ratio (*X*2), and extraction time (*X*3), we used the quadratic multinomial regression equation as follows:

(2) $$\begin{array}{c} Y1=27.32-2.25X1-6.491X2-0.318X3-2.249X1X2+0.690X1X3-1.181X2X3-5.062X1^{2}-2.179X2^{2}-5.990X3^{2}. \end{array}$$

The ANOVA results and regression coefficients are presented in Tables 3 and 4, indicating that the contribution of the quadratic model was significant (*p* < 0.05).

### 3.3. Statistical Analysis and Model Fitting of Polyphenol Content

The polyphenol content of hydroethanolic extract varied from 87.45 to 266.23 mg EAG/g extract based on different extraction conditions (Table 2). Multiple regression analyses of variance (ANOVA) were used to check the adequacy and fitness of the developed models, and the results of ANOVA are given in Table 5. The results showed that the response of the worth rate (*Y*) model was highly significant. The *R*^2^ value was 0.964 (Table 5), indicating that 96.4% of the total variation in the polyphenols content was attributed to the experimental variables studied, namely, ethanol concentration, extraction time, and solvent-to-material ratio. The independent variables, ethanol concentration (*X*1), solvent/material ratio (*X*2), and extraction time (*X*3) are significant (*p* < 0.05). As well as the quadratic variables (*X*1-1, *X*2-2, and *X*3-3) and interaction variable (*X*1*X*2, *X*1*X*3, and *X*2*X*3) are extremely significant (*p* < 0.05) (Table 6).

To calculate the polyphenol content in extract (*Y*2) encoded by independent variables ethanol concentration (*X*1), solvent-to-material ratio (*X*2), and time extraction (*X*3), we used the quadratic multinomial regression equation as follows:

(3) $$\begin{array}{c} Y2=168.83+5.53X1-49.531X2-17.60X3-29.61X1X2+8.81X1X3+6.65X2X3-15.28X1^{2}+13.34X2^{2}+9.30. \end{array}$$

### 3.4. Effect of Extraction Parameters on Polyphenol Content

**Figure 4:**
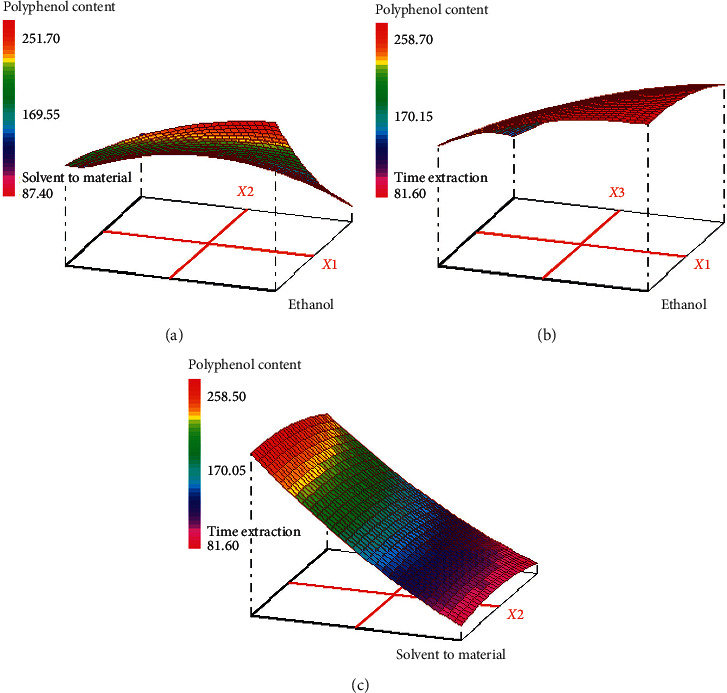
Response surface for the effect of independent variables on polyphenol content in hydroethanolic extract. (a) Response surface graph showing interaction between solvent-to-material ratio (*X*2) and ethanol concentration (*X*1); (b) response surface graph showing interaction between extraction time (*X*3) and solvent-to-material ratio (*X*2); (c) response surface graph showing interaction between extraction time (*X*3) and ethanol concentration (*X*1).

**Figure 5:**
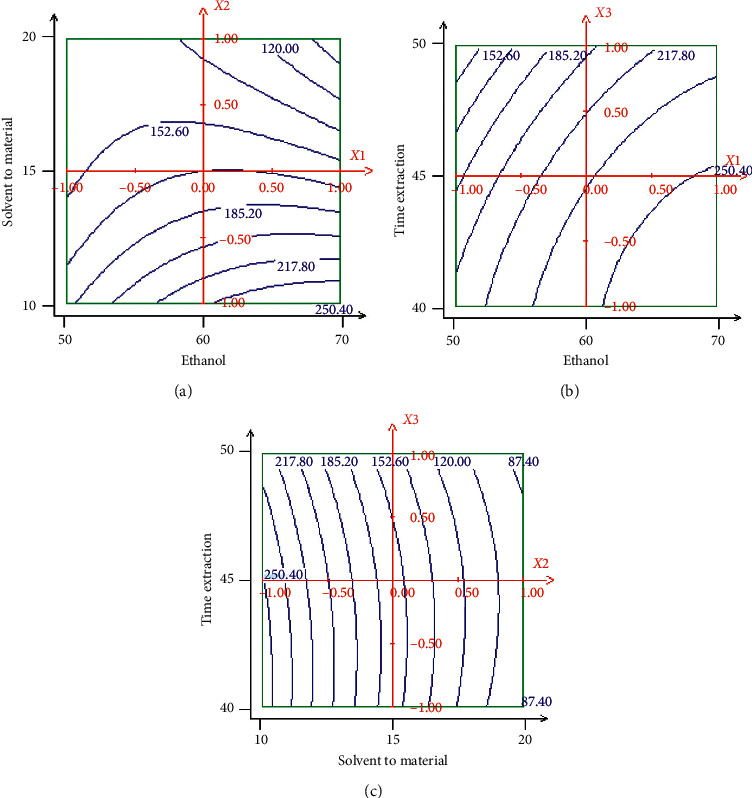
Contour plots for the effect of independent variables on polyphenol content in hydroethanolic extract. (a) Contour plot graph showing interaction between solvent-to-material ratio (*X*2) and ethanol concentration (*X*1); (b) contour plot graph showing interaction extraction time (*X*3) and solvent-to-material ratio (*X*2); (c) contour plot graph showing interaction between extraction time (*X*3) and ethanol concentration (*X*1).

The response surface and contour plots of the effects of extraction parameters on the polyphenol content are presented in Figures 4 and 5. As shown in Figure 4(a), the efficiency of ethanol concentration on the polyphenol content was evaluated and the results showed that the polyphenol content increased with the increase of ethanol concentration (70%). It can be concluded that maximum polyphenol content (257.88 mg EAG/g extract) could be achieved when the ethanol concentration and solvent-to-material ratio were 68 and 10 mL/g, respectively, at the fixed extraction time in 40 min. The polyphenol yield increased with an increase of ethanol concentration from 68% to 70%, while decreased when the ethanol concentration was above 70%. Polyphenol yield was largely dependent on the polarity content of solvents and compounds, and a single solvent may not be effective for the extraction of a bioactive compound. Therefore, a combination of ethanol and water was more effective in extracting phenolic compounds than ethanol alone [31]. 70% of the ethanol showed the highest polyphenol yield, due to lower viscosity and a modification of the plant structure by swelling the matrix, which allowed the solvent to penetrate more completely into the plant material, and these results are in agreement with [32, 33]. Therefore, water acts as a swelling agent for plants, while ethanol would disturb the binding between solutes and plant matrices. Therefore, the mixture of water and ethanol showed the best performance for the extraction of phenolic compounds among all the extracts used; these results are in agreement with [34]. As for the effect of solvent-to-material ratio of the polyphenols yield, the yield increased with increasing the solvent-to-material ratio from 10 to 15 mL/g and decreased after 15 mL/g. The interaction effect of extraction time and ethanol concentration on the polyphenols content is presented in Figure 4(b). It was found that maximum polyphenol content is achieved when the ethanol concentration was 70% and the extraction time was 50 min fixed solvent-to-material ratio at 10 mL/g. The interaction effect of extraction time and solvent-to-material ratio of the polyphenol content, at a fixed ethanol concentration of 70%, is also presented in Figure 4(c). It was found that maximum polyphenol content is achieved when the extraction time was 41 min and the solvent-to-material ratio was 10 mL/g. It is concluded that the ethanol concentration, the solvent-to-material ratio and extraction time have a significant interaction effect.

### 3.5. Effect of Extraction Parameters on Yield Extraction

**Figure 6:**
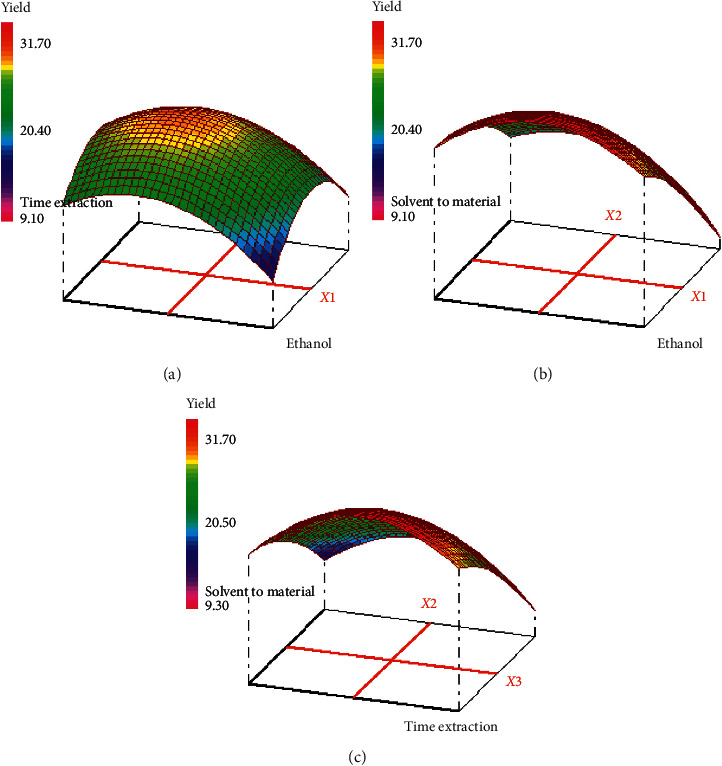
Response surface for the effect of independent variables on extraction yield. (a) Response surface graph showing interaction between extraction time (*X*3) and ethanol concentration (*X*1); (b) response surface graph showing interaction between solvent-to-material ratio (*X*2) and ethanol concentration (*X*1); (c) response surface graph showing interaction between extraction time (*X*3) and solvent-to-material ratio (*X*2).

**Figure 7:**
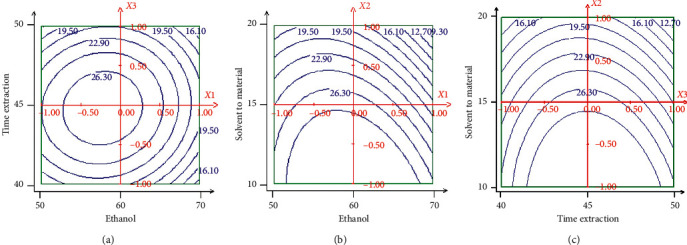
Contour plots for the effect of independent variables on extraction yield. (a) Contour plot graph showing interaction between extraction time (*X*3) and ethanol concentration (*X*1); (b) contour plot graph showing interaction between solvent-to-material ratio (*X*2) and ethanol concentration (*X*1); (c) contour plot graph showing interaction between extraction time (*X*3) and solvent-to-material ratio (*X*2).

In Figures 6(a) and 7(a), when the 3D response surface plot and the contour plot were developed for the extraction yield of hydroethanolic extract, with varying extraction time and ethanol concentration at fixed solvent-to-material 15 mL/g. At a definite ethanol concentration, the yield of extraction decreased slightly with the increase of ethanol concentration and extraction time. The highest extraction yield occurred in ethanol concentration of 57% and extraction time of 45 min [35]. In Figures 6(b) and 7(b), when the 3-D response surface plot and the contour plot were developed for the recovery of hydroethanolic extract with varying ethanol concentration and solvent-to-material ratio at fixed extraction time at 45 min, it can be seen that maximum recovery in yield of extraction can be achieved when ethanol concentration and solvent-to-material ratio were 59% and 15 mL/g, respectively. Figures 6(c) and 7(c) show the effect of the solvent-to-material ratio and extraction time on the extraction yield of hydroethanolic extract at a fixed ethanol concentration of 60%. The yield of hydroethanolic extract was decreasing evidently as the increasing of solvent-to-material ratio and nearly reached a peak at the highest extraction time tested. It can be seen that maximum recovery of hydroethanolic extract can be achieved when plant material-to-solvent ratio and extraction time were around 10 mL/g and 47 min.

### 3.6. Verification of Predictive Models

Applying the methodology of RSM, the optimum level of various parameters was obtained and it indicated that extraction time 40 min, ethanol concentration of 70%, and solvent-to-material ratio of 11 mL/g give a maximum of polyphenols content of 257.86 mg/g and yield extraction 31.37%. These optimal conditions could be considered as optimum as well as feasible conditions. To compare the predicted result with the practical value, the rechecking experiment was performed using some extraction condition. The value obtained from real experiments, demonstrated the validity of the RSM model, since there were no significant (*p* > 0.05) differences (Table 7). The strong correlation between the real and the predicted results confirmed that the response model was adequate to reflect the expected extraction conditions.

## 4. Conclusion

In this study, the optimization of ultrasound-assisted extraction of hydroethanolic extract of *Z. mays* hairs and calculation of their polyphenols content were conducted. Through response surface methodology (RSM) of yield, the optimal conditions were determined as follows: extraction time 40 min, solid-liquid ratio 11 mL/g, and ethanol concentration 70%. Under the optimum conditions, the extraction yield of hydroethanolic extract and polyphenol content was 31.37% and 257.86 mg EAG/g extract, respectively, which allowed higher extraction yields with lower temperature and extraction time when compared with conventional solvent extraction methods. With stable results, this method offered a theoretical basis for industrial and experimental extraction of hydroethanolic extract from *Z. mays* hairs.

## Figures and Tables

**Table 1:** Levels of variables for the experimental design.

Independent variable	Symbol	Coded levels		
		−1	0	1
Ethanol/water (%)	X1	50	60	70
Solvent/material ratio (mL/g)	X2	10	15	20
Time (min)	X3	40	45	50

**Table 2:** Box–Behnken design (coded) arrangement for extraction of *Z. mays* hairs.

N°Exp	Rand	Ethanol (mL)	Solvent to material (mL/g)	Time extraction (min)	Yield (%)	Polyphenol content (Mg EAG/g extract)
1	—	50	10	45	26.35	187.24
2	—	50	10	45	26.34	187.00
3	—	70	10	45	28.23	250.28
4	—	70	10	45	28.19	250.25
5	—	50	20	45	16.47	126.78
6	—	50	20	45	16.44	126.70
7	—	70	20	45	9.32	87.45
8	—	70	20	45	9.33	87.44
9	—	50	15	40	20.12	166.78
10	—	50	15	40	20.11	166.77
11	—	70	15	40	12.34	151.34
12	—	70	15	40	12.34	151.34
13	—	50	15	50	18.83	119.54
14	—	50	15	50	18.82	119.50
15	—	70	15	50	13.81	139.37
16	—	70	15	50	13.81	139.33
17	—	60	10	40	24.45	240.24
18	—	60	10	40	24.44	240.20
19	—	60	20	40	15.24	147.52
20	—	60	20	40	15.22	147.50
21	—	60	10	50	25.45	184.54
22	—	60	10	50	25.44	184.52
23	—	60	20	50	11.51	119.24
24	—	60	20	50	11.50	119.23
25	—	60	15	45	27.34	168.87
26	—	60	15	45	27.31	168.80

**Table 3:** Analysis of variance (ANOVA) for the second-order model correlating the content of phenolic compounds with the experimental variables.

Source	Sum of squares	Degree of freedom	Mean square	*p* value
Regression	1019.57	9	113.28	<0.01^*∗∗∗*^
Residual	24.16	16	1.51	

Validity	24.16	3	8.05	<0.01^*∗∗∗*^
Pure error	0.0225	13	0.00173	

Total	1043.74	25	—	—

*R* ^2^ = 0.977; *R*^2^_Adj_ = 0.964; *R*^2^_pred_ = 0.941.

**Table 4:** Effect estimation, statistical significance, and regression coefficient estimates for phenolic compounds extraction from *Z. mays* hairs.

Source	Coefficient	Degree of freedom	*p* value
*b*0	14.186	—	<0.01^*∗∗∗*^
*X*1	1.450	1	<0.01^*∗∗∗*^
*X*2	0.771	1	0.0451^*∗∗∗*^
*X*3	−4.996	1	<0.01^*∗∗∗*^
*X*1-1	1.178	1	0.0302^*∗∗∗*^
*X*2-2	−0.714	1	0.207^*∗∗*^
*X*3-3	−0.664	1	0.271^*∗∗*^
*X*1-2	0.300	1	4.36^*∗*^
*X*1-3	0.200	1	12.4
*X*2-3	0.192	1	13.5

**Table 5:** Analysis of variance (ANOVA) for the second-order model correlating the content of phenolic compounds with the experimental variables.

Source	Sum of squares	Degree of freedom	Mean square	*p* value
Regression	56823.57	9	6313.73	<0.01^*∗∗∗*^
Residual	2115.23	16	132.20	—
Validity	2115.53	3	705.06	—
Pure error	0.00378	13	0.029	—
Total	5893.74	25	—	—

*R* ^2^ = 0.964; *R*^2^_Adj_ = 0.944; *R*^2^_pred_ = 0.908.

**Table 6:** Effect estimation, statistical significance, and regression coefficient estimates for phenolic compounds extraction from *Z. mays* hairs.

Nom	Coefficient	Sum of squares	Signif. (%)
*b*0	27.325	0.0093026051	<0.01^*∗∗∗*^
*b*1	−2.257	0.0032889676	<0.01^*∗∗∗*^
*b*2	−6.491	0.0032889676	<0.01^*∗∗∗*^
*b*3	−0.318	0.0032889676	<0.01^*∗∗∗*^
*b*1-1	−5.062	0.0061530949	<0.01^*∗∗∗*^
*b*2-2	−2.179	0.0061530949	<0.01^*∗∗∗*^
*b*3-3	−5.990	0.0061530949	<0.01^*∗∗∗*^
*b*1-2	−2.249	0.0046513025	<0.01^*∗∗∗*^
*b*1-3	0.690	0.0046513025	<0.01^*∗∗∗*^
*b*2-3	−1.181	0.0046513025	<0.01^*∗∗∗*^

**Table 7:** Predicted and experimental values of the responses at some extraction conditions.

	Ethanol *X*1 (%)	Extraction time *X*3 (min)	Solid-liquid ratio *X*2	Extraction yield (%)	TPC (mg EGA/g extract)
Optimum conditions	70	40	11	31.37	257.86
Predicted conditions	70	40	11	30.25	255.34

## Data Availability

No data were used to suppor this study.
